# Exploring the RING-Catalyzed Ubiquitin Transfer Mechanism by MD and QM/MM Calculations

**DOI:** 10.1371/journal.pone.0101663

**Published:** 2014-07-08

**Authors:** Yunmei Zhen, Guangrong Qin, Cheng Luo, Hualiang Jiang, Kunqian Yu, Guanghui Chen

**Affiliations:** 1 Department of Biology, Shantou University, Guangdong, China; 2 Drug Discovery and Design Center, State Key Laboratory of Drug Research, Shanghai Institute of Materia Medica, Chinese Academy of Sciences, Shanghai, China; 3 Department of Chemistry, Shantou University, Guangdong, China; Wake Forest University, United States of America

## Abstract

Ubiquitylation is a universal mechanism for controlling cellular functions. A large family of ubiquitin E3 ligases (E3) mediates Ubiquitin (Ub) modification. To facilitate Ub transfer, RING E3 ligases bind both the substrate and ubiquitin E2 conjugating enzyme (E2) linked to Ub via a thioester bond to form a catalytic complex. The mechanism of Ub transfer catalyzed by RING E3 remains elusive. By employing a combined computational approach including molecular modeling, molecular dynamics (MD) simulations, and quantum mechanics/molecular mechanics (QM/MM) calculations, we characterized this catalytic mechanism in detail. The three-dimensional model of dimeric RING E3 ligase RNF4 RING, E2 ligase UbcH5A, Ub and the substrate SUMO2 shows close contact between the substrate and Ub transfer catalytic center. Deprotonation of the substrate lysine by D117 on UbcH5A occurs with almost no energy barrier as calculated by MD and QM/MM calculations. Then, the side chain of the activated lysine gets close to the thioester bond via a conformation change. The Ub transfer pathway begins with a nucleophilic addition that forms an oxyanion intermediate of a 4.23 kcal/mol energy barrier followed by nucleophilic elimination, resulting in a Ub modified substrate by a 5.65 kcal/mol energy barrier. These results provide insight into the mechanism of RING-catalyzed Ub transfer guiding the discovery of Ub system inhibitors.

## Introduction

Protein modifications by the addition of Ub or ubiquitin-like proteins have emerged as major mechanisms that control diverse biological processes not only in the cell [1], but also in developing tissues [2]. The Ub system is implicated in cancer, neurodegenerative disorders inflammatory, and infectious diseases, justifying its study [3]–[7]. Ubiquitylation is achieved by three classes of enzymes, ubiquitin-activating enzymes (E1s), ubiquitin conjugating enzymes (E2s), and ubiquitin ligase enzymes (E3s). E1 forms a covalent thioester intermediate with the C terminus of Ub in an ATP-dependent manner. This activates the Ub transfer process. Ub is transferred from E1 to E2 via a transthiolation reaction resulting in a covalent thioester linkage between E2' catalytic cysteine and Ub's C terminus (E2∼Ub). Finally, E3s promote the transfer of Ub from E2 to the substrate lysine amino group. There are two domains in E3s binding E2 and substrate to facilitate this process. E1 and E3 binding sites on E2s substantially overlap, so E2s dissociate from ligase domains to be “reloaded” with Ub [8]–[10].

E3 ligases dominantly fall into two sub classes bearing either a HECT (homologous to E6-AP carboxyl terminus) domain or a RING (really interesting new gene) domain. HECT-containing E3 proteins with a small number in human are directly involved in catalysis: Ub is transferred from E2 to the catalytic cysteine of the HECT domain, and then to a target lysine residue. RING family members represent most of the E3s, of which there are over 600 encoded in the human genome. They possess a conserved arrangement of cysteine and histidine residues that coordinate two zinc atoms [11]. Unlike HECT E3, RING E3 ligases function as substrate recognition factors and mediate direct transfer of Ub from E2 to a lysine on the substrate or Ub itself, creating a polyubiquitin chain [12], [13].

Small ubiquitin-like modifier (SUMO) modifies proteins post-translationally [14]. SUMO-targeted ubiquitin ligases (STUbLs) are a conserved family of proteins that target SUMO-modified proteins for ubiquitylation [15]–[22] and typified by RING finger protein 4 (RNF4) in mammals [23]. Four SUMO interaction motifs (SIMs) in the N-terminal region of RNF4 allow it to engage polySUMO-modified substrates. A RING domain [11] in the C-terminal region is responsible for dimerization and catalysis of Ub transfer [24], [25]. RNF4 plays a key role in DNA damage response [26]–[30], accurate chromosome segregation during mitosis [31], [32], and arsenic therapy for acute promyelocytic leukaemia [17], . It also regulates the localization and function of the HTLV-1 oncoprotein Tax [34] that promotes cell survival during reduced oxygen conditions [35], as well as other pathophysiological conditions [36], [37].

Ubiquitination can be outlined in the following steps: E2∼Ub changes conformation when bounds to a RING E3 ligase [38], [39]. In this way, the thioester bond linking E2 and Ub is highly activated. An incoming substrate lysine is deprotonated and acts as a nucleophile for the E2∼Ub thioester bond, followed by the substrate binding. How RING E3s promote Ub transfer remains unclear. Three major mechanisms have been proposed for lysine deprotonation: i) a local microenvironment [40] which reduces the substrate lysine pK, ii) the optimal position of the incoming lysine ε-amino group and reactive thioester bond [41], and iii) an acidic residue attracts the proton from the ε-amino group [1], [42]. These suspects are only based on structures and activity analysis from kinetic and mutational biochemical experiments [42]–[46]. No theoretical studies were found to describe intermediates and associated energy barriers in the reaction pathway. Therefore, our endeavors turn to understanding transition state points and reaction energy barriers, which may apply to regulators of ubiquitin ligase enzymes.

Taken together, the elaborate elucidation of ubiquitin transfer catalysis is not only of great fundamental interest, but also of high medical relevance. Thus, we investigated the catalytic mechanism of Ub transfer by combining molecular modeling, MD simulations, and QM/MM calculations. Two different substrate binding models of RNF4 RING-UbcH5A-Ub-SUMO2 (E3-E2-Ub-substrate) in aqueous solution were obtained from an MD simulation. The most proper model was chosen to probe the catalytic mechanism of proton transfer and nucleophilic attack. Our simulation results highlight the role of residues D117 and N77, which are consistent with experiment studies. These findings provide an atomic description of Ub transfer including mechanisms for substrate lysine deprotonation and nucleophilic attack.

## Materials and Methods

### Preparation of the simulation system

The initial configuration of the enzyme-substrate complex was modeled on the crystal structures of RNF4 RING-UbcH5A-Ub (E3-E2-Ub, PDB code: 4AP4) [42] and SUMO–RanGAP1–Ubc9–RanBP2 structure (Ubl-substrate-E2-E3, PDB code: 3UIP) [47], and solution structure of SUMO2 (substrate, PDB code: 2AWT). There are 20 different conformations for SUMO2 in solution, and the first conformation was chosen to build the initial model. Firstly, the UbcH5A∼Ub (E2∼Ub) thioester model was generated from the crystal structure by replacing K85 on E2 UbcH5A with a cysteine using Sybyl software package (Tripos, St. Louis, MO). Since E2 UbcH5A and E2 Ubc9 are homologous proteins, E2 UbcH5A of the RNF4 RING–UbcH5A–Ub (E3-E2-Ub) thioester complex was superimposed onto E2 Ubc9 in the SUMO–RanGAP1–Ubc9–RanBP2 (Ubl-substrate-E2-E3) complex. Finally, the substrate SUMO2 was superimposed onto substrate RanGAP1 in the SUMO–RanGAP1–Ubc9–RanBP2 (Ubl-substrate-E2-E3) complex using PyMOL. As K11 is the main nucleophilic attack lysine embedded in the conservative (I/V/L)Kx(D/E) motif [48], ten sequential residues nearby the conservative motif were used for superposition. Two models were achieved by superposition with different sequence segments (every sequence segment includes the conservative motif).

The resulting RNF4 RING-UbcH5A-Ub-SUMO2 complexes were energy minimized using Sybyl via the Tripos force field with the following parameters: amber charges, distance-dependent dielectric function, and nonbonded cutoff of 8 Å for the protein. The structures were minimized by the simplex method first, followed by the Powell method to an energy gradient of 0.05 kcal/(mol·Å).

### Molecular dynamics simulations

Before MD simulations, protonation states of ionizable residues in the complex were determined using the H++ program [49] and manually checked according to local electrostatic and hydrogen bond microenvironments. For the K11 on substrate SUMO2, two protonation states were considered. The charges and force field parameters for the four zinc finger domains were established from a previous study [50]. The charge information for glycine and cysteine connected through a thioester bond was calculated using the RESP method [51] encoded in the AMBER suite of programs (version 10.0) [52] at the HF/6-31G* level. The complex system was reloaded into AMBER after applying the AMBER Parm99 force field,and solvated into a octahedral box of TIP3P [53] water molecules with a water thickness extending 12 Å away from the protein surface. Finally, energy minimization of the solvated system was demonstrated.

All MD simulations were performed using the AMBER package (version 10.0) under constant temperature and pressure (NPT) and periodic boundary conditions. The Amber99SB [54]–[56] force field and TIP3P model were applied to protein and water molecules, respectively. Electrostatic interactions were calculated using the particle-mesh Ewald method [57], while bond lengths involving hydrogen atoms were constrained with the SHAKE algorithm [58] during MD simulations. An integration step of 2 fs was adopted. For nonbonded interactions, a cutoff radius of 10 Å was employed. The nonbonded pair was updated every 25 steps. Each simulation was applied by the Berendsen algorithm [59], coupling the protein to a 300 K thermal bath at 1.0 atm of pressure (1 atm  = 101.3 kPa). The temperature and pressure coupling parameters were set at 1 ps.

### QM/MM calculations

QM/MM calculations were performed using a two-layer ONIOM [60] method encoded in the Gaussian09 program [61]. The complex is divided into two regions: the “odel” system, in which bond breaking and formation occurs and is treated with the most accurate (high-level) method; and the “real” system, treated with inexpensive (low-level) model chemistry corresponding to environmental effects of the molecular environment on the “model” system. The total ONIOM extrapolated energy is defined as [62]:
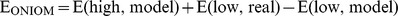
E (high, model) represents the energy of the model system (including link atoms) with the high accuracy method; E (low, real) is the energy of the real system at the low level of theory, and E (low, model) stands for the energy of the model system at the low level of theory. Therefore, the ONIOM method allows for high-level calculation on a small but critical part of the system and merges surrounding effects at a lower level of theory to yield an energy expression for the entire system. As a result, accuracy in computational complexity is reduced by performing high level calculation on a small region.

In the present study, initial coordinates for QM/MM calculations were extracted from MD simulations. The QM system contains critical catalytic residues. In the process of proton transfer, the QM system consisted of 44 atoms, including G76 on Ub, C85 and D117 on E2 UbcH5A and the K11 on substrate SUMO2. For nucleophilic attack, the QM system contained 98 atoms, including G75 and G76 from Ub, C85, N77, N114, D117, P118 and L119 on E2 UbcH5A, and K11 from SUMO2. Other atoms of the complex were classified into the MM system. Besides, QM boundaries were treated with the link atom approach [63] by introducing hydrogen atoms to saturate the valence of QM boundary atoms. The QM region was described by density functional theory (DFT) with the hybrid Becke three-parameter Lee-Yang-Parr exchange-correlation functional (B3LYP) [64] and 6-31G* basis set [65] for favorable computational effort/accuracy ratio. The AMBER Parm99 force field was used for MM calculation. A reaction energy profile was evaluated by single point energy calculations at the B3LYP QM/MM level.

## Results and Discussion

It was of vital importance to obtain an accurate model for the enzyme-substrate complex. The initial RNF4 RING-UbcH5A-Ub-SUMO2 (E3-E2-Ub-substrate) complex structure was modeled on the basis of three structures with high resolution. The model was then refined based on information from existing crystal structures and experimental data. Dynamic conformational changes were studied through MD simulations. Results were carefully compared to biochemical data for validation. Subsequent QM/MM simulations were performed to investigate the mechanism of Ub transfer to scan the potential energy for interactions between the substrate and enzyme throughout the process. The lysine deprotonation process before substrate nucleophilic attack was also investigated using the same protocol.

### The binding models of the RNF4 RING–UbcH5A–Ub-SUMO2 complex

To investigate the molecular mechanism of Ub transfer, we built a model for the RNF4 RING–UbcH5A–Ub-SUMO2 (E3-E2-Ub-substrate) thioester complex. The initial configuration of the enzyme-substrate complex was modeled on the basis of the crystal structure of RNF4 RING-UbcH5A-Ub (E3-E2-Ub, PDB code: 4AP4) [42] and SUMO–RanGAP1–Ubc9–RanBP2 (Ubl-substrate-E2-E3, PDB code: 3UIP) [47], and solution structure of SUMO2 (substrate, PDB code: 2AWT). The structure of RNF4 RING-UbcH5A-Ub (E3-E2-Ub) includes a dimeric RING domain of E3 RNF4 in complex with E2 UbcH5A linked by an isopeptide bond to Ub. The RNF4 RING-UbcH5A-Ub (E3-E2-Ub) structure shows that Ub in the Ub∼E2 thioester is in a fold-back conformation in which its I44 hydrophobic patch engages the α2 helix of E2 UbcH5A, an intermediate that catalyzes Ub transfer [47], [66]–[68]. The folded-back conformation was evident for SUMO–RanGAP1–Ubc9–RanBP2 (Ubl-substrate-E2-E3), and represented the binding mode between the E3-E2-Ub thioester and substrate. Therefore, the superimposing of E2 Ubc9 from the SUMO–RanGAP1–Ubc9–RanBP2 (Ubl-substrate-E2-E3) and E2 UbcH5A from the RNF4 RING-UbcH5A-Ub (E3-E2-Ub) generated a model of the catalytic transfer complex [42], [69]. E3 RNF4 provides substrate selectivity, and SUMO2 is widely used as the typical substrate [33], [42].

The substrate lysine is the nucleophilic attack group in the Ub transfer system. SUMO2 contains eight lysine residues, and previous reports identified SUMO2 to be ubiquitylated at K11, K32 and K41 *in vivo* [33]. K11 is the most abundant Ub linkage [70] and major internal SUMO2 acceptor site for SUMO poly-conjugation [71]. Hence, K11 was chosen as the nucleophilic attack lysine to probe the transfer mechanism.

As mentioned in Materials and Methods, the E2 ligase superposition of RNF4 RING–UbcH5A–Ub (E3-E2-Ub) thioester and SUMO–RanGAP1–Ubc9–RanBP2 (Ubl-substrate-E2-E3) formed a model for the RNF4 RING–UbcH5A–Ub-SUMO2 (E3-E2-Ub-substrate) complex with 1.04 Å root-mean-square deviations (RMSD). This indicated that the superposition was reliable. From substrate superposition, ten sequential residues including the Ψ-K-x-D/E motif from the two substrates met well, resulting in two different models with the same RMSD 1.31 Å named R1 and R2. So, the process of superposition substrate was reasonable. The resulting models with coordinates conflicting or missing superposition of K11 were excluded. Finally, two proper models were achieved by the superposition of E8-H17(SUMO2/2AWT)---G521-K530(RanGAP1/3UIP) and V10-N19(SUMO2/2AWT)---L523-I532(RanGAP1/3UIP), respectively, named R1 and R2 (Figure S1). The two complex structures were verified for use as initial structures for subsequent MD and QM/MM studies.

To obtain a more reasonable RNF4 RING-UbcH5A-Ub-SUMO2 (E3-E2-Ub-substrate) complex for mechanism discussion, 35 ns MD simulations were performed on the two models. RMSD was monitored to detect model stability during the whole MD process (from t = 0 ps) using backbone coordinates of the model structures as reference. For R1, RMSD is flat after 13 ns simulation shown in Figures S2A and S2B. The K11 on SUMO2 moves away from the active region of RNF4 RING–UbcH5A–Ub (E3-E2-Ub) thioester complex. The minimum distance between the N_ε_ of K11 of SUMO2 and carbonyl carbon (C_-CO-_) of G76 on E2 UbcH5A is 5.74 Å beyond the attack distance, indicating that this binding model is unsuitable to address the Ub transfer process. For the R2 model simulation system, the RMSD is flat after 13 ns simulation indicated that this area's structures are more favorable (Figure S2C, S2D and S3). The distance between N_ε_ of K11 and C_-CO-_ of G76 is kept within the 2.87 Å to 9.01 Å range, prompting the proposed nucleophilic attack. RNF4 functions in dimeric form. The initial model build by the way described in the Materials and Methods may create fake interface between RNF4 and Ub because the two RING domains lose one binding partner. So we provide a superposition between equilibrated model from the MD simulation and start conformation (crystal structure) shown in Figure S4. Structure comparison shows they meet well except the substrate SUMO2. The interactions between the RNF4 RING and Ub of the equilibrated model are similar to that in the crystal structure. So the model we build is reasonable. We thus only continue with the discussion of the R2 model.

### MD Simulation Analysis for RNF4 RING–UbcH5A–Ub–SUMO2 complex

**Figure 1:**
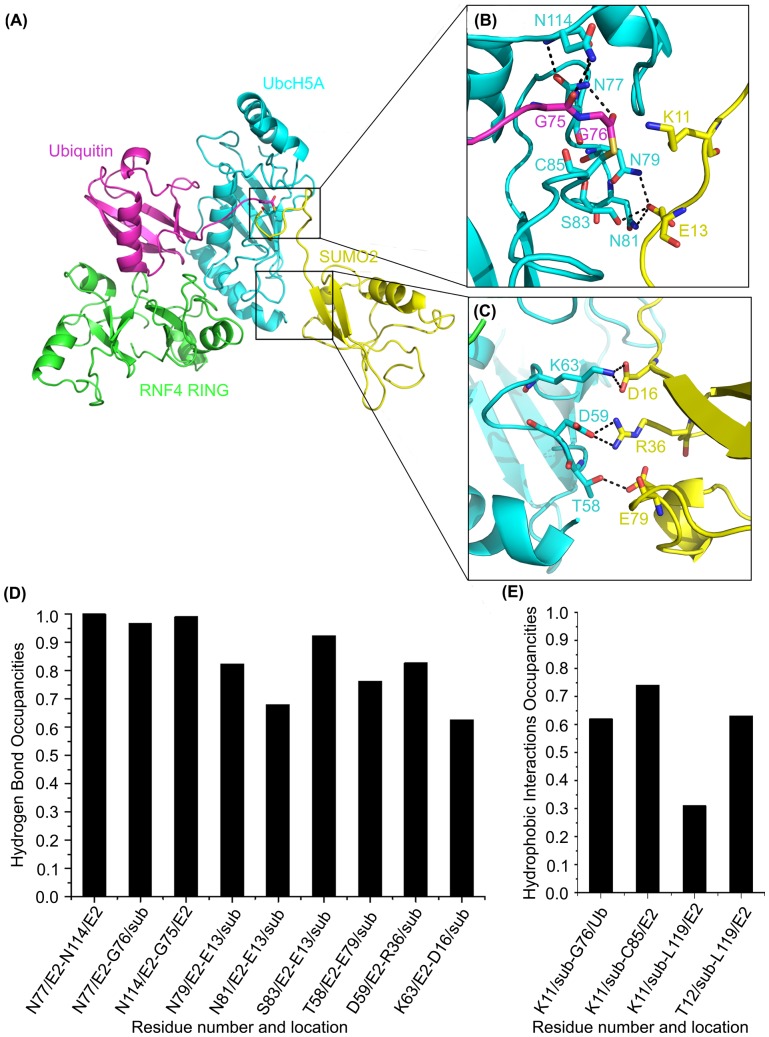
Interactions between UbcH5A (E2) and SUMO2 (sub) in the R2 trajectory. E2 UbcH5A is shown in cyan, E3 RNF4 is shown in green, Ub is shown in magenta, and substrate SUMO2 is shown in yellow. (A–C) Detail of interactions between E2 UbcH5A (cyan) and substrate SUMO2 (yellow). (D) Hydrogen bonds occupancies during production MD. (E) Hydrophobic interactions occupancies during production MD.

Residues located in the H-bond network are commonly validated experimentally for their critical roles in protein stability and catalytic processes, and can thus offer important evidence for interactions influencing binding affinity. For the R2 simulation system, the three H-bonds (N77/E2-N114/E2, N77/E2-G76/sub and N114/E2-G75/E2) in the active site are conserved after the system equilibrated shown as Figure 1. This provides additional evidence that stable H-bond interactions maintain the thioester in reactive conformation. The importance of N77 and N114 was verified by mutagenesis studies. N77A of E2 UbcH5A completely abolished catalytic activity and N114A of E2 UbcH5A modestly reduced activity [38]. Moreover, N77, a key residue in the catalytic HPN motif located upstream of the active site cysteine residue, stabilizes an oxyanion intermediate formed within an E2∼Ub/Ubl thioester during the substrate lysine nuclephillic attack [40], [68], [72]. Residues N79, N81 and S83 on E2 UbcH5A form three H-bonds with E13 on SUMO2, tightly anchoring the loop of substrate to E2. Moreover, T58, D59 and K63 on E2 UbcH5A are important for substrate binding. The distance between T58 and substrate E79 is within H-bond distance. D59 hydrogen bonds with the substrate's D36. K63 forms a hydrogen bond with D16 on substrate. Thus, this network contributes to the tight binding of E2 enzyme UbcH5A and substrate SUMO2, further mediating the Ub reaction process.

Hydrophobic interactions also play important roles to stabilize the substrate in the active site. As mentioned before, the substrate VKTE motif directly contacts the catalytic chamber. In the R2 model, nearly all residues in the catalytic chamber are involved in hydrophobic interactions with the substrate including G76 on Ub and C85 and L119 on E2 UbcH5A, emphasizing the importance of each residue in the catalytic chamber for precise lysine side chain positioning. This result is consistent with mutational analysis [42]. Remarkably, the L119 side chain, positioned on the opposite side of the E2 catalytic cleft, interacts with substrate K11 and T12, implying its fundamental role on positioning the substrate in a productive binding orientation for catalysis. This synergy stabilizes the catalytic environment and facilitates the biochemical reaction via structural and electrostatic effects. Taken together, consistence between the simulation and experimental data demonstrates that MD simulations of RNF4 RING–UbcH5A–Ub-SUMO2 (E3-E2-Ub-substrate) were reasonable and the model was reliable for further study.

### Nucleophilic attack in ubiquitylation

The stable binding model of substrate on the E2 enzyme triggers ubiquitylation. Here, QM/MM calculations were carried out to draw the mechanism of nucleophilic attack in ubiquitylation. Starting structures for QM/MM calculations were sampled from the MD trajectory of the R2 model. When choosing the initial structure for QM/MM calculation, two criteria were considered: the position of the side chain of K11 in substrate (orientating to the active site of RNF4 RING-UbcH5A-Ub (E3-E2-Ub) complex), and proper distances for atoms involved in nucleophilic attack (distances between N_ε_ of K11 on substrate SUMO2 and C_-CO-_ of G76 on Ub, S of C85 on E2 UbcH5A were less than 3.5 Å). Furthermore, based on the RMSD during the simulations, two representative structure snapshots at 20.370 ns and 20.582 ns were extracted from the MD trajectory and selected as initial structures for subsequent QM/MM studies. For QM layer atoms selection, those residues which proved to be critical for nucleophilic attack were considered. These included G75, G76 on Ub, C85, N77, N114, D117, P118, and L119 on E2 UbcH5A and K11 on substrate SUMO2 (Figure S5). The two structures showed a similar trend with differences only in energy barriers. Further QM/MM discussion is focused on the energetically favorable 20.37 ns structure.

The QM/MM optimized geometry of the reagent system shown in Figure S5 varied slightly with the initial structure obtained from MD simulations. The side chain of K11 on substrate SUMO2 lay in front of the thioester bond. The distance from N_ε_ of K11 on SUMO2 to C_-CO-_ of G76 on Ub relaxed from 3.32 Å to 3.49 Å. The angle formed by G76 main chain carbonyl and K11 Nϵ, namely A (O = C---N_ε_), increased from 112.72° to 128.25°. Distances from N_ε_ of K11 on SUMO2 to oxygen atoms of the D117 carboxyl and P118 main chain on E2 UbcH5A were respectively 3.56 Å and 3.10 Å, indicating that H-bonds were important in the catalytic center. The optimized configuration was quite compatible with the stereochemistry principles for the reaction, demonstrating that the model was reliable for QM/MM calculations.

**Figure 2:**
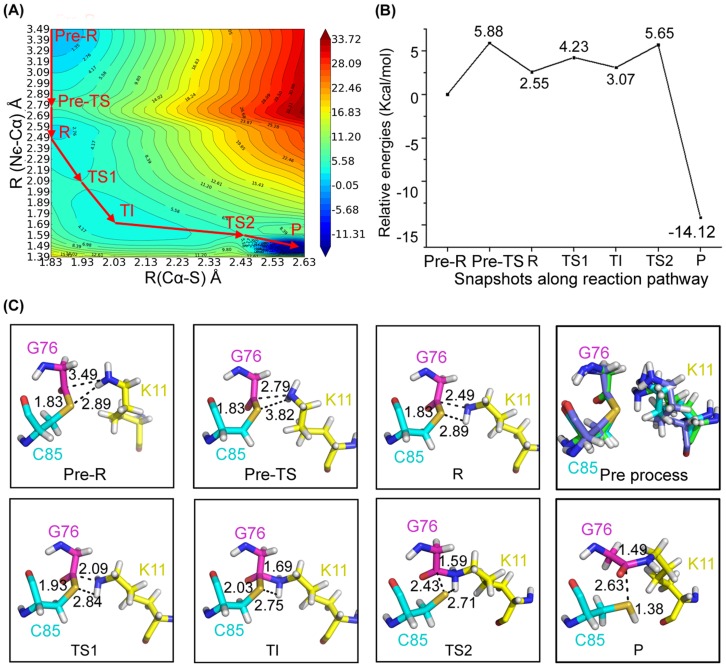
Overview of the nucleophilic attack. (A) The potential energy surface of nucleophilic attack along defined reaction coordinates. (B) The relative energy of representative conformations along the reaction pathway. (C) The QM/MM optimized structures of the reactant (R), transition states (TSs), transition immediate (TIs), and product (P).

By calculating the two-dimensional QM/MM potential energy surface (PES) with reaction distance coordinates of R (N_ε_-C_-CO-_) and R (C_-CO-_-S), an optimized pathway was determined. Along the optimized pathway, several representative states were observed (Figure 2). Two steps including K11 reorientation and nucleophilic reaction were characterized from analyzing the energy and structures extracted from PES.

Before the incoming lysine nucleophilic attack thioester bond between E2 and Ub, the side chain of the incoming lysine from substrate undergoes conformation change to approach the complex's active region. In this step, N_ε_ of K11 from substrate approaches the carbonyl carbon of G76 on Ub, while the thioester bond remains unchanged. Movement of K11 from substrate induces the rupture of the two H-bonds between K11 and D117, P118. Moreover, the two hydrogen atoms of the NH2 group in K11 from substrate rotate to ensure N_ε_ of K11 get close to the reaction center to form pre-TS state. These processes induce relative energy barrier of 5.88 kcal/mol. With further rotation of the hydrogen atoms, K11 is ready for nucleophilic attack resulting in the reagent (R state) with relatively energy of 2.55 kcal/mol. The optimal orientation and proximity of K11 may be an essential preparation measure for the subsequent nucleophilic attack.

The resulting R state which contributed by the above preparation step is ready for nucleophilic attack, so it is treated as reactant structure with the N_ε_-C_-CO-_ bond R (N_ε_-C_-CO-_)  = 2.49 Å and C_-CO_-S bond R (C_-CO-_-S)  = 1.83 Å. With the decrease of R (N_ε_-C_-CO-_) and increase of R (C_-CO-_-S), the thioester bond is attacked by lysine, forming a transition immediate. As shown in Figure S6, in the optimized transition immediate, the N_ε_-C_-CO-_ bond is formed (R (N_ε_-C_-CO-_)  = 1.69 Å) and the C_-CO_-S bond is elongated (R (C_-CO-_-S)  = 2.03 Å). Meanwhile, the distance between N_D2_ of N77 on E2 UbcH5A and O of G76 on Ub is 2.89 Å and the angle consisted of N_D2_-H-O is 147.65°, which indicate the amino group of N77 interacts with the thioester carbonyl to stabilize the anionic (oxyanion) intermediate. Additionally, the H75 on UbcH5A interacts with N77 with the distance of imidazole ring N_D1_ and main chain of nitrogen R (N_D1_-N)  = 3.33 Å and the angle of the hydrogen bond A (N_D1_-H-N)  = 165.39° to regulate the electronic nature of the amide group, which also would contribute to oxyanion hole stabilization. To be consistent with our calculations, mutations of the H75 and N77 on E2 led to decrease in reactivity [40], [42], [73], [74]. These observations also show that electrostatic complementarity is one of the key functions for E2, which agrees with former investigation [43]. From the curve plotted in Figure 2B, the energy barrier needed for nucleophilic attack is 4.23 kcal/mol. The structure of TS1 is determined by adiabatic mapping at the QM/MM level. For TS1, R (N_ε_-C_-CO-_) equals 2.09 Å and R (C_-CO-_-S) equals 1.93 Å. The dimethylamino group tends to be in a plane concomitant with the contraction of the C_M_-N_T_ bond. Structural reorganization clearly illustrates that the N_ε_-C_-CO-_ bond is partially formed while the Cα-S bond is partially broken.

Similarly, the energy shift of the TI-P process was also monitored as the highest point of the reaction path. In the optimized product structure, R (N_ε_-C_-CO-_) equals 1.49 Å and R (C_-CO-_-S) equals 2.73 Å. The proton of the K11 side chain amino group is transferred to S of C85, inducing the separation of Ub and UbcH5A, through the structure of TS2 with R (N_ε_-C_-CO-_)  = 1.59 Å and R (C_-CO-_-S)  = 2.43 Å. The energy barrier of the process is 5.65 kcal/mol, while the product relative energy equals to -14.12 kcal/mol, which is much less than the reactant, suggesting the stability of product.

Low energy barriers in the Ub transfer process and low energy of the final state indicate this process is energetically favorable. Results of the QM/MM simulation, especially the TS and TI structures, may offer us further insight on the discovery of regulators against E3.

### Deprotonation of lysine from the substrate

Before nucleophilic attack, another important characteristic of K11 deprotonation should be considered for lysine is usually positively charged in physiological environment. There had been controversy about the deprotonation process of K11 on substrate. The most possible deprotonation process is catalyzed by an acidic residue. The Lys11-specific E2 Ube2S which lacks an acidic residue in its active site utilizes the Glu34 of the acceptor Ub for Lys11 activation [41]. Experiment data also proved that the acidic residue D117 on E2 positions and/or activates the incoming lysine [42]. Therefore, we also studied the process of lysine deprotonation. The conformation used for QM/MM calculation in nucleophilic attack was used as the initial structure for further 35 ns MD simulations with a protonated K11, particularly an R2_H model. In this model, the K11 on SUMO2 was charged.

**Figure 3:**
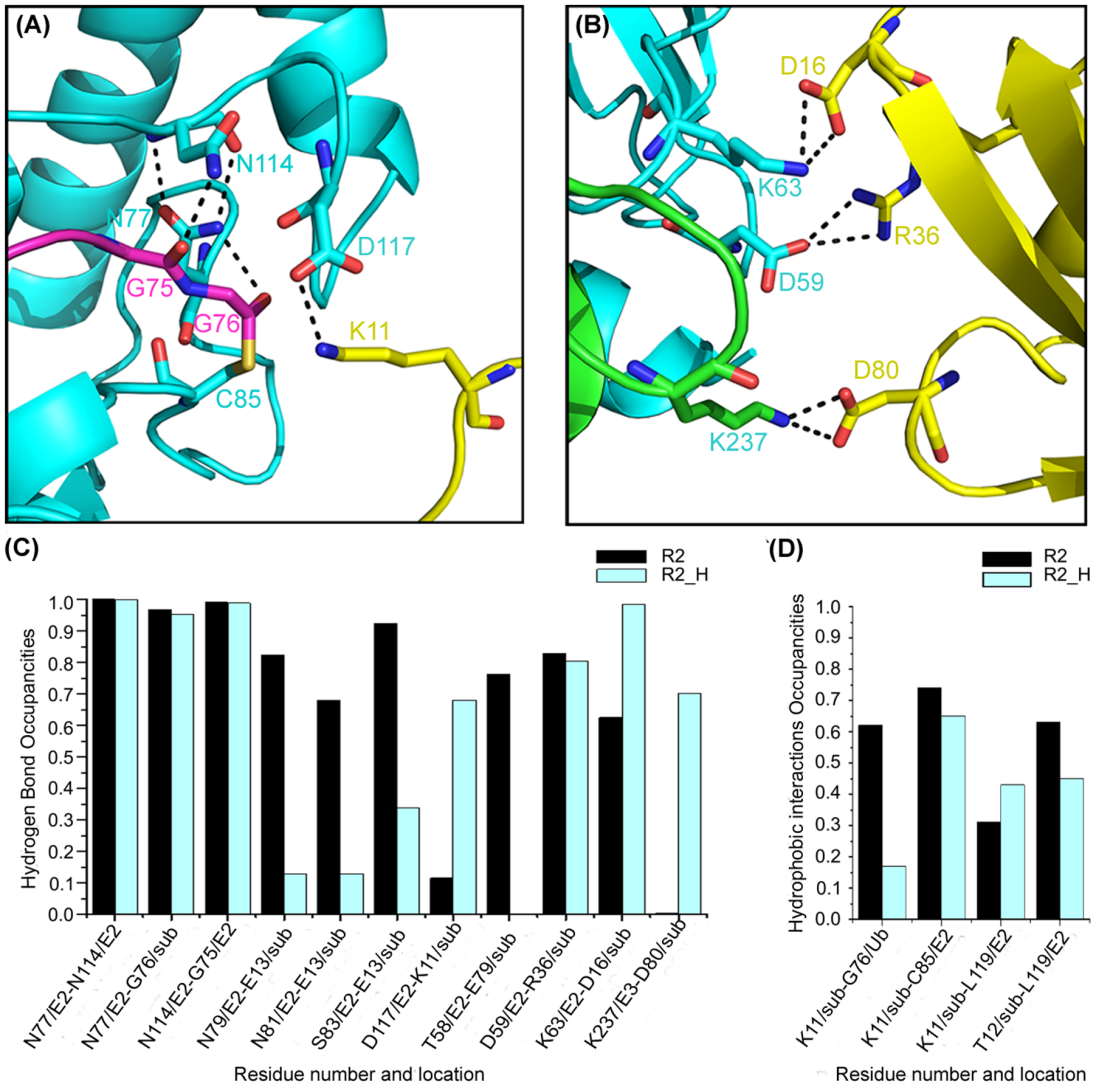
Interactions between enzyme (E2 and E3) and substrate (sub) in the R2_H trajectory and comparison of the R2 system. E2 UbcH5A is shown in cyan, E3 RNF4 is shown in green, Ub is shown in magenta, and substrate SUMO2 is shown in yellow. (A) Structure detail of the interaction between E2 (UbcH5A) and substrate (SUMO2) in the E2 active site. (B) Detail of the substrate (SUMO2) and ligase interaction. (C) Hydrogen bond occupancies in R2_H compared to R2. (D) Hydrophobic interaction occupancies of both R2 and R2_H systems.

The RMSD of the C-alpha atoms tends to be flat after 13 ns simulation and the active region fluctuate within 2 Å as shown in Figure S2, which indicated that structures located in this area theoretically are more reasonable. The H-bonds (N77-N114, N77-G76 and N114-G75) in the active site were similar to the model of R2 (Figure 3). The H-bonds of D59/E2-R36/Ub and K63/E2-D16/Ub are similar to those in model R2. The hydrogen bonds formed by E13 and residues in E2 enzyme significantly decreased in the R2_H system, as the side chain of E13 on substrate SUMO2 moved far away from E2. K11 on substrate SUMO2 was tightly linked to D117 along the trajectory, indicating D117 in E2 functions to position and/or active the incoming lysine. T58/E2-E79/Ub H-bond is lost in the R2-H model. As a complement, K237 on E3 RNF4 interacts with substrate SUMO2 via D80, which stabilizes the substrate binding. Compared to the R2 model, hydrophobic interactions make little difference, except the interaction between K11 from the substrate and G76 on Ub. When K11 is positively charged, it is unstable at the reaction center, initiating movement around D117 on E2 UbcH5A along the MD trajectory. These also indicated that the deprotonation of K11 is an essential preparation step for ubiquitylation.

**Figure 4:**
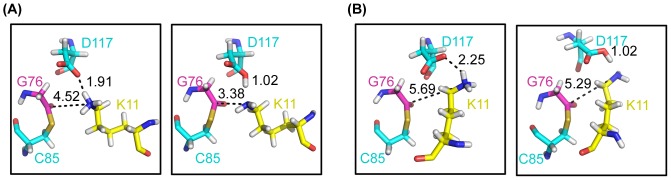
Two possible deprotonation pathways of lysine through D117. (A) Average structure (A′) in the first cluster in R2_H MD simulations and its optimized conformation (A″). (B) The second clustered structure (B′) and its optimized conformation (B″).

Within 5 Å of the N_ε_ of K11, there are two acidic residues: D117 on E2 UbcH5A and E13 on substrate SUMO2. Through the analysis of the MD trajectory and hydrogen bonding, D117 may be in better position to obtain the proton from the incoming lysine. K11 on SUMO2 which has a long side chain moves around D117 on UbcH5A along the MD trajectory. To study the deprotonation process using QM/MM, we clustered the active region (G76 on Ub, C85 and D117 on E2, and V10-E13 on substrate) into two optimal conformations. The starting structures for QM/MM calculations were the average conformations of the two clusters shown in Figure 4. The main difference between the two clusters is the position of K11 relative to D117 on E2 UbcH5A: one is between D117 and the thioester bond and the other is on the opposite side of D117. The QM region is composed of D117 on E2 UbcH5A and K11 from SUMO2. The remainder of the complex is included in the MM region.

After the representing structures of the two clusters were optimized, the proton of the positively charged K11 on SUMO2 transferred to the carboxyl oxygen (O_D2_) of D117 on E2 enzyme with no energy barrier. For the first cluster, the distance between the carboxyl oxygen (O_D2_) of D117 and hydrogen (H_Z3_) of K11 changed from 1.9 Å to 1.0 Å, and the incoming lysine moved toward the thioester bond. The distance between N_ε_ of K11 and C_-CO-_ of G76 was 3.38 Å, favorable for nucleophilic attack as shown in the above section. For the second cluster, the incoming lysine is positioned around D117 on E2 UbcH5A, but far away from the thioester bond of the active region. Similar with the conformation from the first cluster, the hydrogen (H_Z3_) of K11 on SUMO2 bonded with the carboxyl oxygen (O_D2_) of D117 on E2 UbaH5A, rupturing the N_ε_-H of the incoming lysine. As lysine's pKA is over 10 in aqueous solution, it easily obtains a proton to be positively charged. In both conformations, K11 is deprotonated through D117, but a direct nucleophilic attack can happen from the first cluster conformation.

## Conclusions

**Figure 5:**
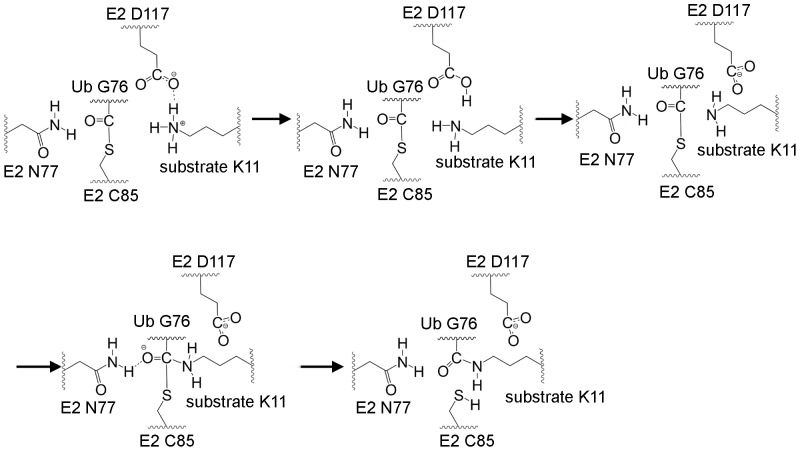
The proposed RING-catalyzed Ub transfer mechanism.

Protein modeling, molecular dynamics and QM/MM calculations were carried out to investigate the RING-catalyzed Ub transfer reaction from E2 to substrate. The constructed model of RING–substrate–UbcH5A–Ub thioester complex and following MD simulations obtained reliable conformations to investigate the mechanism of ubiquitylation. An integral ubiquitylation mechanism was concluded. The Ub transfer process is triggered through the covalent thioester bonding of Ub and E1. The active Ub transfers from E1 to E2 forming an E2∼Ub complex. In the final step, lysine from the substrate nucleophilic attacks the thioester bond of E2∼Ub facilitated by RING E3 (Figure 5). The charged lysine in the substrate rotates its side chain to a proper position after substrate binding, preparing for the reaction. D117 on E2 may attract the charged lysine to reach its reaction position. When the amino group of the positively charged lysine moves between the thioester bond in E2∼Ub and D117 on E2, it transfers a proton to D117 easily, forming a deprotonated lysine ready for further nucleophilic attack. The side chain of the deprotonated lysine then undergoes slight conformational change when approaching the thioester bond, facilitating closeness between the N_ε_ and hydrogen atoms of the lysine amino and between the carbonyl carbon and sulfur atoms in E2∼Ub. The N_ε_ of lysine performs a nucleophilic attack on the carbonyl carbon of the thioester bond, resulting in an oxyanion intermediate via TS1 that is stabilized by N77 and H75 on E2. Eventually, the thioester bond breaks and obtains a proton from lysine to release Ub-modified substrates.

As the Ub system plays a vital role in metabolism, DNA damage response, and oxidation systems, the Ub mechanism may be helpful for understanding a large number of biological processes. The present study provides a detailed mechanism for RING-catalyzed Ub transfer, elucidates the molecular basis for this reaction, RING-catalyzed Ub transfer, and may shed light on the discovery of novel mechanism-based regulators of the ubiquity system. Furthermore, results from the present study offer theoretical basis for mechanistic studies on other Ub transfer proteins.

## Supporting Information

Figure S1 **Two initial substrate binding models of RING-substrate-UbcH5A-Ub complex.** E3 RNF4 is shown in green, E2 UbcH5A is shown in cyan, Ub is shown in magenta in both models. Substrate SUMO2 is shown in gray in R1 model and yellow in R2 model.
(DOCX)

Figure S2 **The RMSD of backbone atoms for three models of RNF4 RING-SUMO2-UbcH5A-Ub during the 35 ns simulation.** (A–E) RMSD of the trajectory compared to the initial coordinates in the production run. (B–F) RMSD of the trajectory after 13 ns compared to coordinates at 13ns.
(DOCX)

Figure S3 **The RMSD of backbone atoms for the active region of R2 model during the 13-to-35 ns simulation.** The active region means 8 Å around the thioester bond.
(DOCX)

Figure S4 **Comparison between equilibrated conformation and start conformation of R2 model.** In the start conformation, E3 RNF4 is shown in green, E2 UbcH5A is shown in cyan, Ub is shown in magenta, and substrate SUMO2 is shown in yellow. In the equilibrated conformation, E3 RNF4 is shown in lime, E2 UbcH5A is shown in deepteel, Ub is shown in violet, and substrate SUMO2 is shown in yelloworange. (A) Structure comparison between the two conformations. The largest change is the location of the structured part of SUMO2. (B) Detail of interactions between E3 RNF4 (green) and Ub (magenta) in the start conformation. (C) Detail of interactions between E3 RNF4 (lime) and Ub (violet) in the equilibrated conformation of R2 model. The interactions are similar to that in the start conformation, and the H-bonds in the interface are stable with the occupancy over 85%.
(DOCX)

Figure S5 **The QM/MM optimized structure of Pre-R in the R2 model.** The atoms in the QM region are shown in sticks. E2 UbcH5A is shown in cyan, E3 RNF4 is shown in green, Ub is shown in magenta, and substrate SUMO2 is shown in yellow.
(DOCX)

Figure S6 **The QM/MM optimized structure of TI and key interactions.** E2 UbcH5A is shown in cyan, E3 RNF4 is shown in green, Ub is shown in magenta, and substrate SUMO2 is shown in yellow.
(DOCX)
