# Supplementary material for: Exploring the RING-Catalyzed Ubiquitin Transfer Mechanism by MD and QM/MM Calculations
Source: PLoS One. 2014 Jul 8;9(7):e101663. doi: 10.1371/journal.pone.0101663 (PMC4086935; doi:10.1371/journal.pone.0101663)
Supplement: Figure S1 — Two initial substrate binding models of RING-substrate-UbcH5A-Ub complex. E3 RNF4 is shown in green, E2 UbcH5A is shown in cyan, Ub is shown in magenta in both models. Substrate SUMO2 is shown in gray in R1 model and yellow in R2 model. (DOCX) [file pone.0101663.s001.docx]

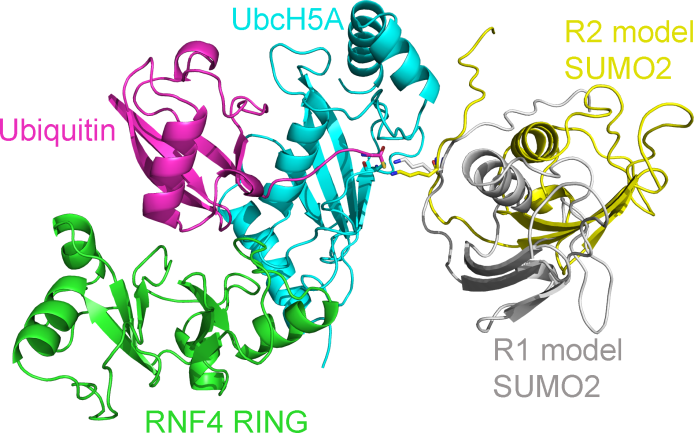


Figure S1. Two initial substrate binding models of RING-substrate-UbcH5A-Ub complex. E3 RNF4 is shown in green, E2 UbcH5A is shown in cyan, Ub is shown in magenta in both models. Substrate SUMO2 is shown in gray in R1 model and yellow in R2 model.
