# Supplementary material for: Exploring the RING-Catalyzed Ubiquitin Transfer Mechanism by MD and QM/MM Calculations
Source: PLoS One. 2014 Jul 8;9(7):e101663. doi: 10.1371/journal.pone.0101663 (PMC4086935; doi:10.1371/journal.pone.0101663)
Supplement: Figure S2 — The RMSD of backbone atoms for three models of RNF4 RING-SUMO2-UbcH5A-Ub during the 35 ns simulation. (A–E) RMSD of the trajectory compared to the initial coordinates in the production run. (B–F) RMSD of the trajectory after 13 ns compared to coordinates at 13ns. (DOCX) [file pone.0101663.s002.docx]

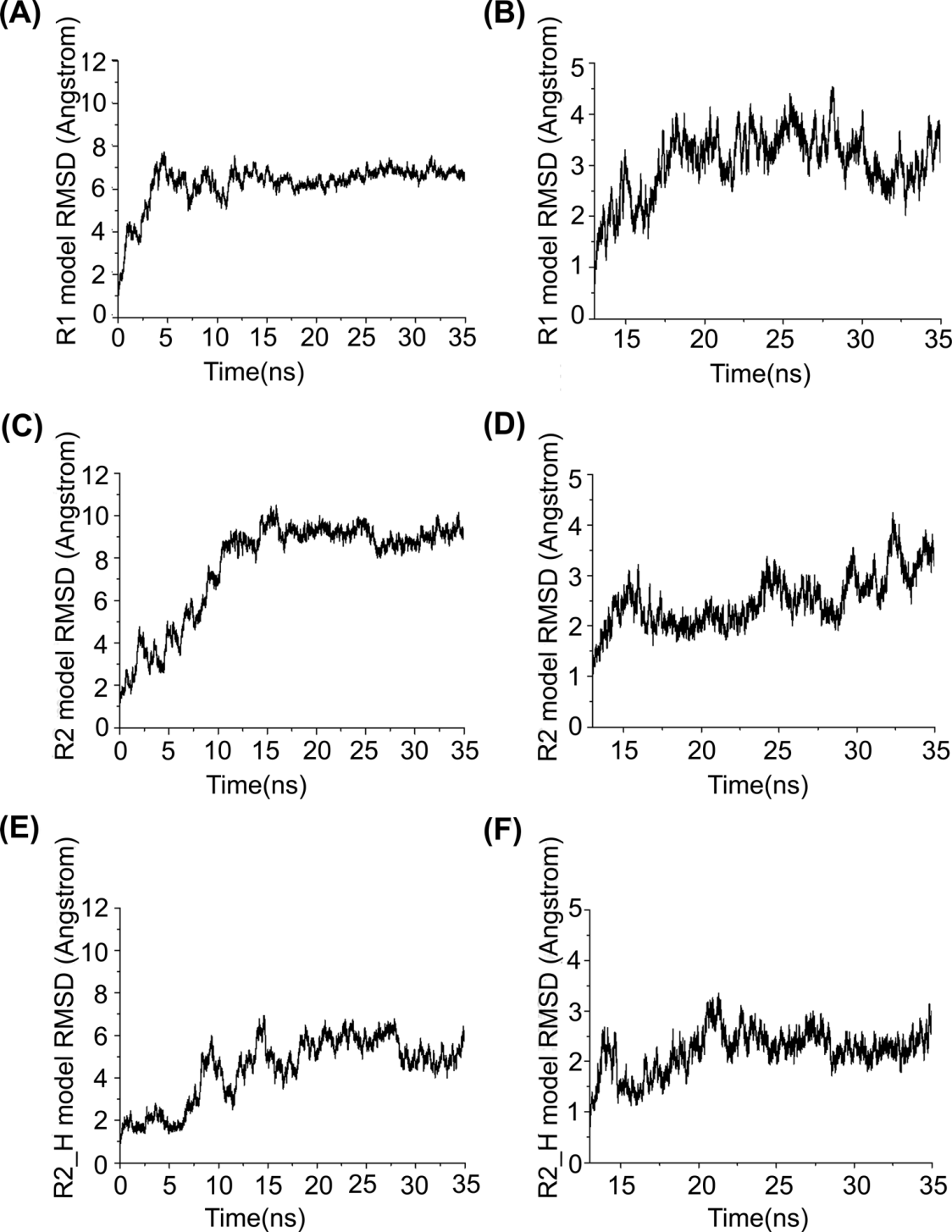


Figure S2. The RMSD of backbone atoms for three models of RNF4 RING-SUMO2-UbcH5A-Ub during the 35 ns simulation. (A-E) RMSD of the trajectory compared to the initial coordinates in the production run. (B-F) RMSD of the trajectory after 13 ns compared to coordinates at 13ns.
