# Supplementary material for: Exploring the RING-Catalyzed Ubiquitin Transfer Mechanism by MD and QM/MM Calculations
Source: PLoS One. 2014 Jul 8;9(7):e101663. doi: 10.1371/journal.pone.0101663 (PMC4086935; doi:10.1371/journal.pone.0101663)
Supplement: Figure S3 — The RMSD of backbone atoms for the active region of R2 model during the 13-to-35 ns simulation. The active region means 8 Å around the thioester bond. (DOCX) [file pone.0101663.s003.docx]

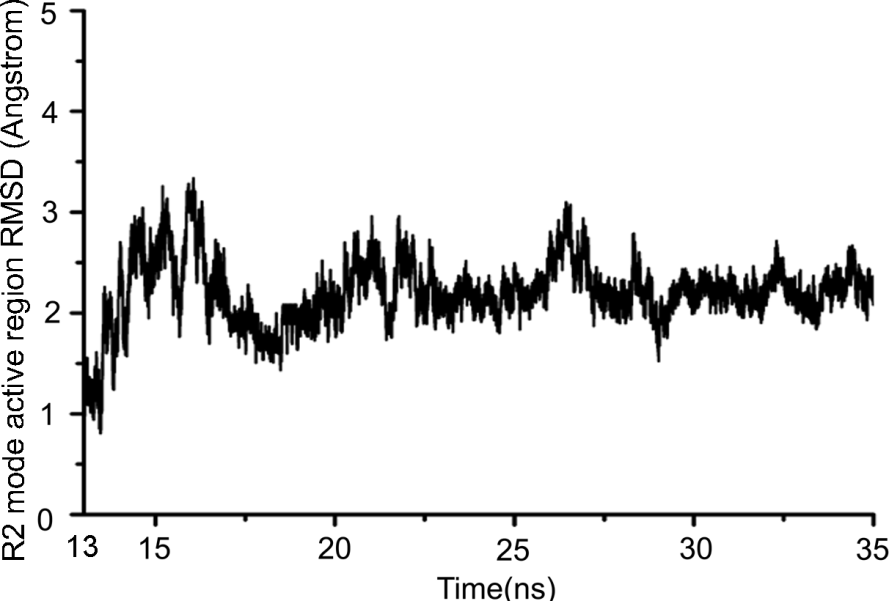


Figure S3. The RMSD of backbone atoms for the active region of R2 model during the 13-to-35 ns simulation. The active region means 8 Å around the thioester bond.
