# Supplementary material for: Exploring the RING-Catalyzed Ubiquitin Transfer Mechanism by MD and QM/MM Calculations
Source: PLoS One. 2014 Jul 8;9(7):e101663. doi: 10.1371/journal.pone.0101663 (PMC4086935; doi:10.1371/journal.pone.0101663)
Supplement: Figure S4 — Comparison between equilibrated conformation and start conformation of R2 model. In the start conformation, E3 RNF4 is shown in green, E2 UbcH5A is shown in cyan, Ub is shown in magenta, and substrate SUMO2 is shown in yellow. In the equilibrated conformation, E3 RNF4 is shown in lime, E2 UbcH5A is shown in deepteel, Ub is shown in violet, and substrate SUMO2 is shown in yelloworange. (A) Structure comparison between the two conformations. The largest change is the location of the structured part of SUMO2. (B) Detail of interactions between E3 RNF4 (green) and Ub (magenta) in the start conformation. (C) Detail of interactions between E3 RNF4 (lime) and Ub (violet) in the equilibrated conformation of R2 model. The interactions are similar to that in the start conformation, and the H-bonds in the interface are stable with the occupancy over 85%. (DOCX) [file pone.0101663.s004.docx]

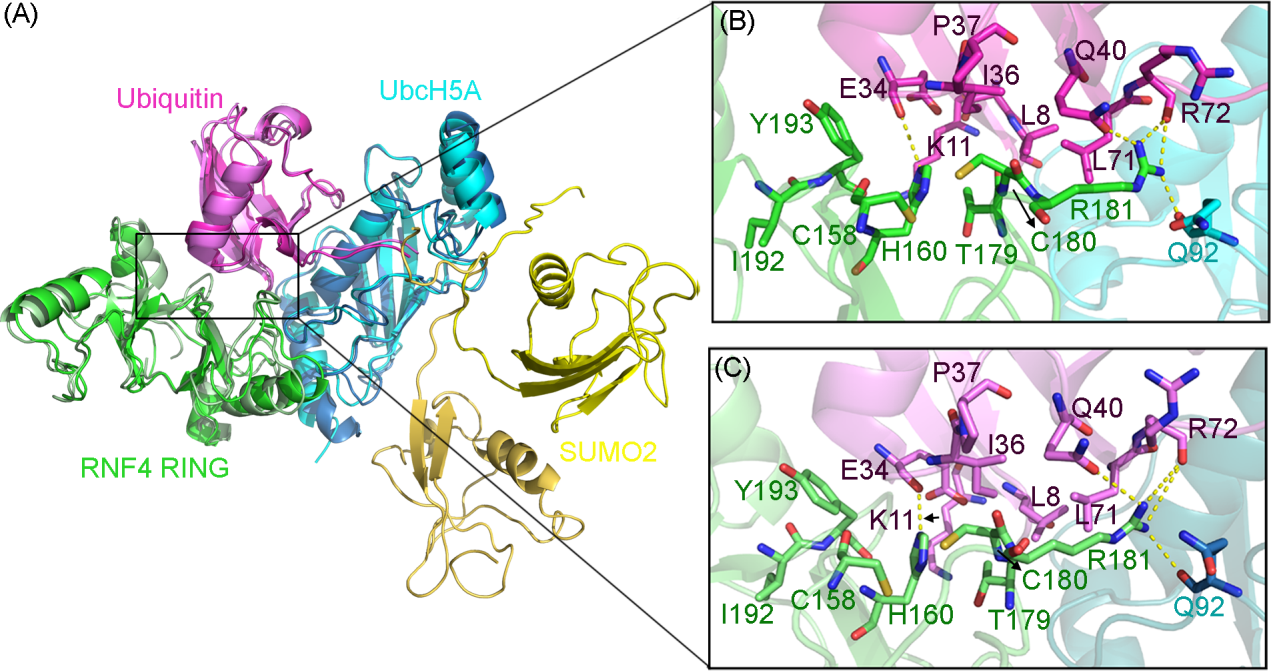


Figure S4. Comparison between equilibrated conformation and start conformation of R2 model. In the start conformation, E3 RNF4 is shown in green, E2 UbcH5A is shown in cyan, Ub is shown in magenta, and substrate SUMO2 is shown in yellow. In the equilibrated conformation, E3 RNF4 is shown in lime, E2 UbcH5A is shown in deepteel, Ub is shown in violet, and substrate SUMO2 is shown in yelloworange. (A) Structure comparison between the two conformations. The largest change is the location of the structured part of SUMO2. (B) Detail of interactions between E3 RNF4 (green) and Ub (magenta) in the start conformation. (C) Detail of interactions between E3 RNF4 (lime) and Ub (violet) in the equilibrated conformation of R2 model. The interactions are similar to that in the start conformation, and the H-bonds in the interface are stable with the occupancy over 85%.
