# Supplementary material for: Exploring the RING-Catalyzed Ubiquitin Transfer Mechanism by MD and QM/MM Calculations
Source: PLoS One. 2014 Jul 8;9(7):e101663. doi: 10.1371/journal.pone.0101663 (PMC4086935; doi:10.1371/journal.pone.0101663)
Supplement: Figure S5 — The QM/MM optimized structure of Pre-R in the R2 model. The atoms in the QM region are shown in sticks. E2 UbcH5A is shown in cyan, E3 RNF4 is shown in green, Ub is shown in magenta, and substrate SUMO2 is shown in yellow. (DOCX) [file pone.0101663.s005.docx]

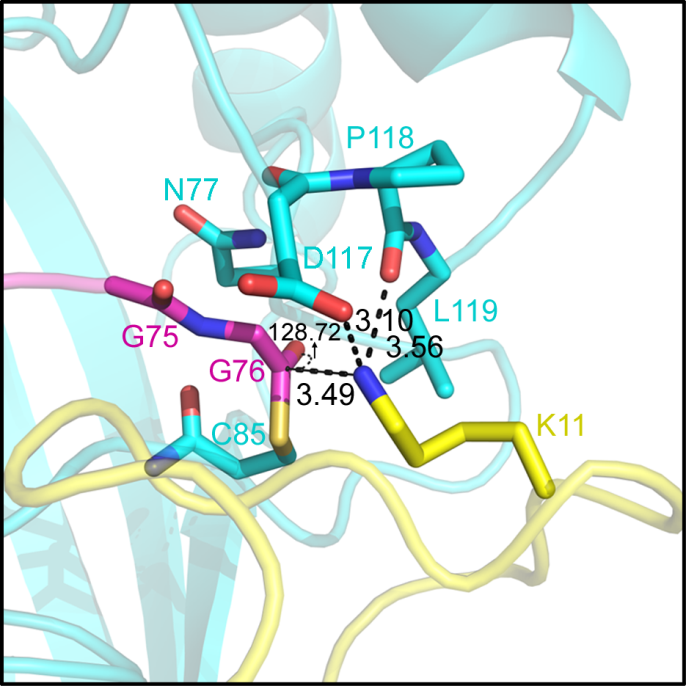


Figure S5. The QM/MM optimized structure of Pre-R in the R2 model. The atoms in the QM region are shown in sticks. E2 UbcH5A is shown in cyan, E3 RNF4 is shown in green, Ub is shown in magenta, and substrate SUMO2 is shown in yellow.
