# Supplementary material for: Exploring the RING-Catalyzed Ubiquitin Transfer Mechanism by MD and QM/MM Calculations
Source: PLoS One. 2014 Jul 8;9(7):e101663. doi: 10.1371/journal.pone.0101663 (PMC4086935; doi:10.1371/journal.pone.0101663)
Supplement: Figure S6 — The QM/MM optimized structure of TI and key interactions. E2 UbcH5A is shown in cyan, E3 RNF4 is shown in green, Ub is shown in magenta, and substrate SUMO2 is shown in yellow. (DOCX) [file pone.0101663.s006.docx]

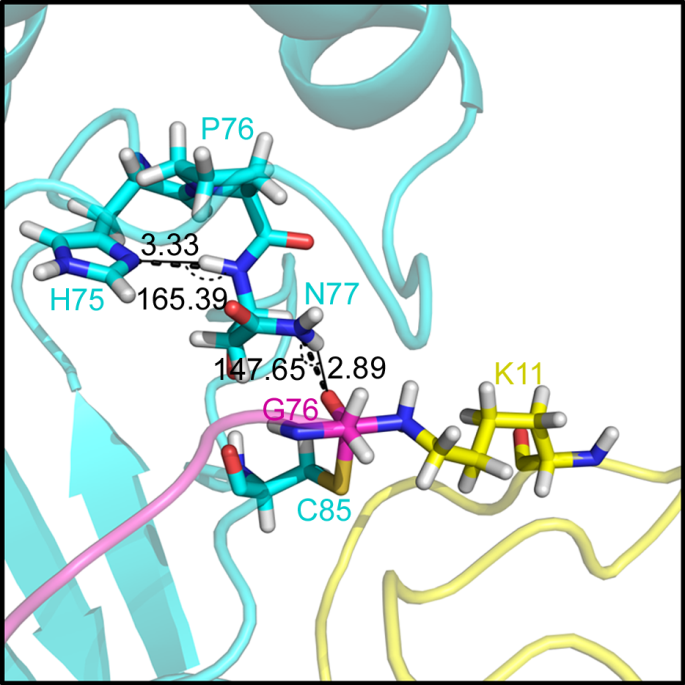


Figure S6. The QM/MM optimized structure of TI and key interactions. E2 UbcH5A is shown in cyan, E3 RNF4 is shown in green, Ub is shown in magenta, and substrate SUMO2 is shown in yellow.
